# Potential role of the gut microbiota of bumblebee *Bombus pyrosoma* in adaptation to high-altitude habitats

**DOI:** 10.3389/fmicb.2023.1218560

**Published:** 2023-08-02

**Authors:** Zhengyi Zhang, Yulong Guo, Mingsheng Zhuang, Fugang Liu, Zhongyan Xia, Zhihao Zhang, Fan Yang, Huayan Zeng, Yueguo Wu, Jiaxing Huang, Jilian Li

**Affiliations:** ^1^State Key Laboratory of Resource Insects, Institute of Apicultural Research, Chinese Academy of Agricultural Science, Beijing, China; ^2^Shanghai Suosheng Biotechnology Co., Ltd., Shanghai, China; ^3^Luoping Yunling Bee Industry and Trade Co., Ltd., Qujing, Yunnan, China

**Keywords:** gut microbiota, functional evolution, high-altitude, energy metabolism, immunity

## Abstract

The gut microbiota affects the health and overall fitness of bumblebees. It can enhance the host's ecological range by leveraging their metabolic capacities. However, the diversity of the gut microbiota and adaptive functional evolution in high-altitude regions remain unclear. To explore how the gut microbiota helps the host adapt to high-altitude environments, we analyzed the differences in diversity and function of the gut microbiota between high- and low-altitude regions through full-length 16S rRNA sequencing. Our results show that high-altitude regions have a lower abundance of *Fructobacillus* and *Saccharibacter* compared to low-altitude regions. Additionally, some individuals in low-altitude regions were invaded by opportunistic pathogens. The gut microbiota in high-altitude regions has a greater number of pathways involved in “Protein digestion and absorption” and “Biosynthesis of amino acids,” while fewer carbohydrate pathways are involved in “digestion and absorption” and “Salmonella infection.” Our finding suggests that plateau hosts typically reduce energy metabolism and enhance immunity in response to adverse environments. Correspondingly, the gut microbiota also makes changes, such as reducing carbohydrate degradation and increasing protein utilization in response to the host. Additionally, the gut microbiota regulates their abundance and function to help the host adapt to adverse high-altitude environments.

## 1. Introduction

Animals harbor gut microorganisms that have evolved along with host lineages, including humans, chimpanzees, gorillas, and orangutans. This provides evidence for the co-diversification of hosts and some lineages of the gut bacteria, implying a long-term vertical association (Moeller et al., 2016; Moran et al., 2019). Similarly, in social corbiculate bees (honey bees, bumble bees, and stingless bees), five core lineages of the gut microbiota show phylogenies mostly matching their hosts, supporting co-diversification over approximately 80 million years (Kwong et al., 2017). The five core gut bacteria, *Gilliamella, Snodgrassella, Bifidobacterium, Lactobacillus Firm-4*, and *Lactobacillus Firm-5*, have undergone functional evolution to adapt to the host. By utilizing their own metabolic capabilities, the gut microbiota help hosts expand their ecological range, which benefits them (Kwong and Moran, 2016). A previous study has indicated that symbiotic bacteria in insects are essential for host health by contributing to food digestion, detoxifying toxic molecules, providing essential nutrients, and protecting against pathogens and parasites (Engel and Moran, 2013; Zheng et al., 2016; Mockler et al., 2018; Nishida and Ochman, 2018).

Bumblebees are important pollinators of wild plants and crops. China has ~50% of the world's species of bumblebees and is the country with the richest bumblebee species (Huang and An, 2018). China is known for its abundant and distinct species of bumblebees, such as *Bombus pyrosoma*, which is found across a wide range, stretching from western Liaoning to eastern Qinghai provinces, and has also been reported in provinces such as Shanxi, Hebei, and Gansu (An et al., 2008; Wu et al., 2009). Their habitats span different ecosystems, from low elevations in the North China Plain to high elevations in the Qinghai–Tibet Plateau (An et al., 2014; Williams et al., 2016). Bumblebees prefer cooler climates, leading to greater diversity in the temperate habitats of the Northern Hemisphere, particularly across Eurasia. In contrast, bumblebee species inhabiting tropical regions tend to be restricted to higher elevations due to the more favorable conditions found there (Engel and Rasmussen, 2020).

High-altitude areas such as the Qinghai-Tibetan Plateau are often accompanied by extreme weather conditions of low temperature, hypoxia, and strong ultraviolet radiation, which are usually unsuitable for the growth and development of biological organisms (Yu et al., 2023). However, organisms such as mammals and ruminants have evolved some strategies to adapt to high-altitude environments (Friedrich and Wiener, 2020). One strategy for dealing with low oxygen levels is to increase oxygen availability in mammals (Storz et al., 2010). For example, populations at high altitudes have lower ventilation than populations at low altitudes (Brutsaert, 2007). In addition, oxygen affinity is enhanced by increasing red blood cell count and hemoglobin level (Weber, 2007). Another strategy is to reduce the body's need for oxygen by lowering its overall metabolic rate (Hochachka et al., 1996). Studies have shown that the energy expenditure of native Andean llamas is significantly lower compared to other ruminants (Riek et al., 2019). High-altitude areas not only affect mammals but also their symbiotic microbiota. Compared to low-altitude individuals, high-altitude species such as humans, Tibetan sheep, and pigs have different compositions and diversity of their symbiotic microbiota (Yang et al., 2017; Ma et al., 2019). However, the metabolic role of the gut microbiota in animals living in high-altitude environments and the potential co-evolution between gut microbiota and host in high-altitude adaptation have not yet been fully understood.

The interaction between gut microbiota not only regulates the organism's health but also forms a crucial bridge between the environment and the host, helping the host to better adapt to the environment (Gao et al., 2020). The unique habitat conditions and simple gut patterns of *Bombus pyrosoma* provide an excellent opportunity to study how gut microbiota help the host adapt to high-altitude evolutionary pressures (Zhang and Zheng, 2022). We hypothesize that the composition and abundance of gut microbiota from *Bombus pyrosoma* in high-altitude areas may differ and help the host improve its ability to adapt to high-altitude environments.

## 2. Materials and methods

### 2.1. *B. pyrosoma* sample collection and DNA extraction

**Figure 1:**
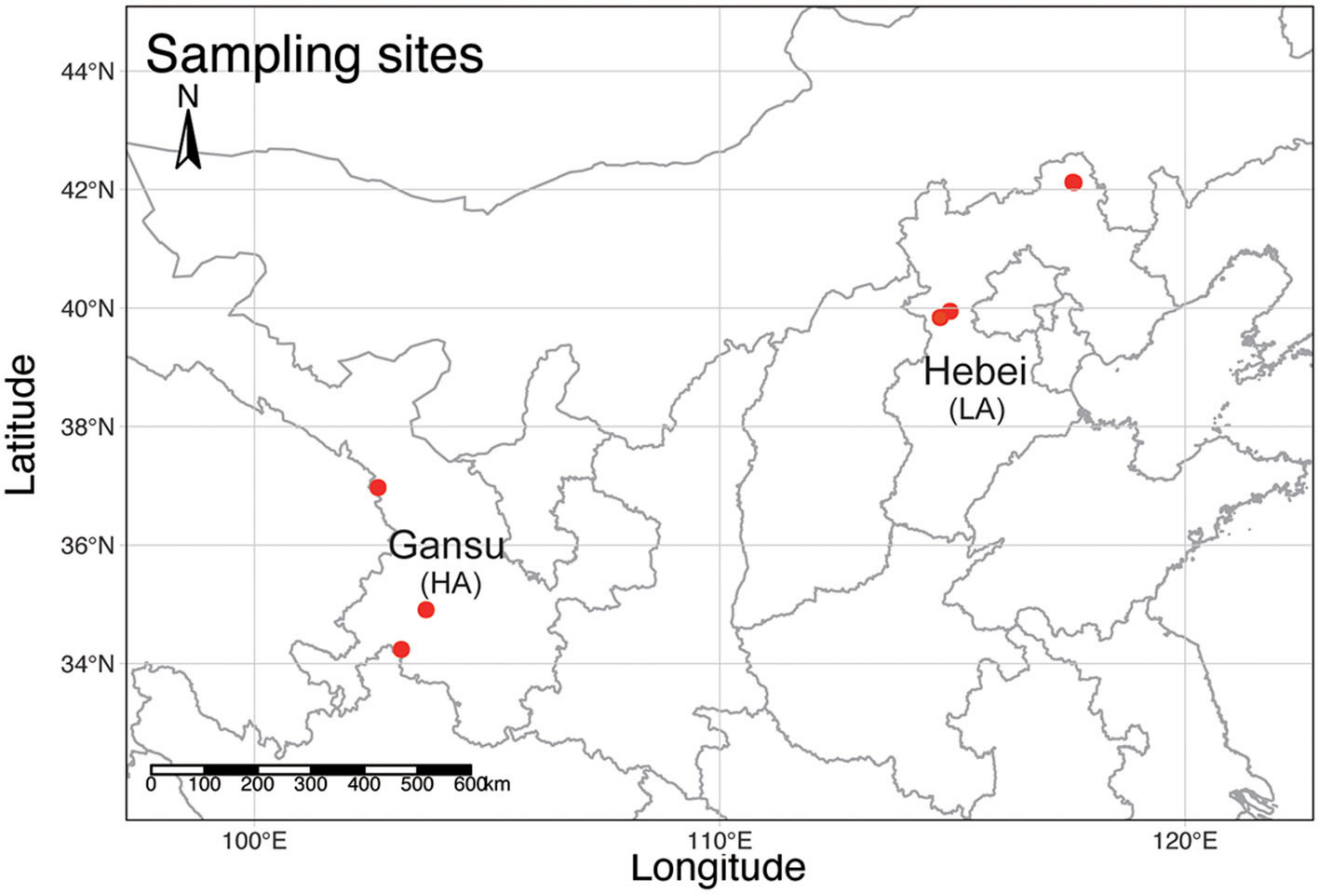
A topographic map of mainland China shows the sampling sites (red dots) of *B. pyrosoma* in this study. Three sites in Gansu represent *B. pyrosoma* individuals at high altitudes (HA, *n* = 21), with each site containing four to ten worker bees as replicate samples. Another three sites in Hebei represent *B. pyrosoma* individuals at low altitudes (LA, *n* = 20), with each site containing five to eight worker bees as replicate samples. The map was created with R (v 4.1.1).

A total of 41 workers of *B. pyrosoma* were collected from six different sites in September 2019 (Figure 1). Three sites in Gansu belong to the high-altitude (HA, *n* = 21) Tibet Plateau (103°9′29.79″, 34°14′23.92″, 3,269 m, 102°39′24.12″, 36°58′14.77″, 2,942 m, 103°41′21.48″, 34°54′34.06″, 2,918 m), with each site containing four to ten worker bees as replicate samples. Another three sites in Hebei belong to the low-altitude (LA, *n* = 20) North China Plain (114°57′5.40″, 39°56′42.04″, 1,192 m, 117°35′25.22″, 42°7′26.50″, 1,027 m, 117°36′53.70″, 42°6′54.95″, 1,005 m) with five to eight replicate samples. The gut DNA of 41 workers of *B. pyrosoma* was extracted according to the protocol of the Wizard^®^ Genomic DNA Purification Kit (Item number: A1125, Promega, Madison, Wisconsin, USA) (Paulos et al., 2016). DNA extraction was dissolved in 30 ml Tris-EDTA (TE) buffer, quantified using a NanoDrop 2000 UV-visible (UV-vis) spectrophotometer (NanoDrop, DE, USA), and qualitatively evaluated by gel electrophoresis.

### 2.2. Full-length 16S rRna sequencing

PCR amplification of the nearly full-length bacterial 16S rRNA genes was conducted using the forward primer 27F (5′-AGRGTTYGATYMTGGCTCAG-3′) and the reverse primer 1492R (5′-RGYTACCTTGTTACGACTT-3′). The PCR reaction conditions were 95°C for 5 min, 95°C for 30 s, 50°C for 30 s, and 72°C for 1 min, with 25 cycles in a reaction volume of 10 μl. A total of PCR amplicons was purified with Agencourt AMPure Beads (Beckman Coulter, Indianapolis, IN) and quantified using the PicoGreen dsDNA Assay Kit (Invitrogen, Carlsbad, CA, USA). After the individual quantification step, amplicons were pooled in equal amounts, and Single Molecule Real Time (SMRT) sequencing technology was performed using the PacBio Sequel platform at Beijing Personal Biotechnology Co., Ltd. (Beijing, China). PacBio circular consensus sequencing (CCS) reads were derived from the multiple alignments of sub-reads to decrease the sequencing error rate.

### 2.3. Bioinformatics and statistical analysis

We used the complete DADA2 workflow (https://github.com/benjjneb/LRASManuscript) to obtain amplicon sequence variants (ASVs) and feature abundance from PacBio CCS reads (Callahan et al., 2019). The obtained ASVs were taxonomically annotated in the SILVA 138 Database (Quast et al., 2013). The calculation of alpha diversity and beta diversity was based on the randomly extracted 4,530 sequencing abundances per sample. Analysis of similarities (ANOSIM) and permutational multivariate analysis of variance (PERMANOVA) were performed with R (v 4.1.1) scripts (Liu et al., 2021). PICRUSt2 (Douglas et al., 2020) contained the Kyoto Encyclopedia of Genes and Genomes (KEGG) orthologs (KO) database and was performed to predict categories and the abundance of Kyoto Encyclopedia of Genes and Genomes (KEGG) pathways (Kanehisa et al., 2012). Specifically, we first used PICRUSt2 to predict KO with the sequence and abundance of ASVs as input and then converted KO to KEGG pathway categories by executing scripts. In addition, we used LDA effect size (LEfSe) (Chang et al., 2022) to conduct different taxonomic hierarchical analyses and STAMP (Parks et al., 2014) for functional differential analysis. The Kruskal–Wallis test was conducted to test the difference in variables (e.g., alpha diversity and KEGG pathways) of gut microbiota found in high-altitude and of those found in low-altitude areas.

## 3. Results

### 3.1. Diversity differences in the gut microbiota between bumblebees in high-altitude and those in low-altitude areas

**Figure 2:**
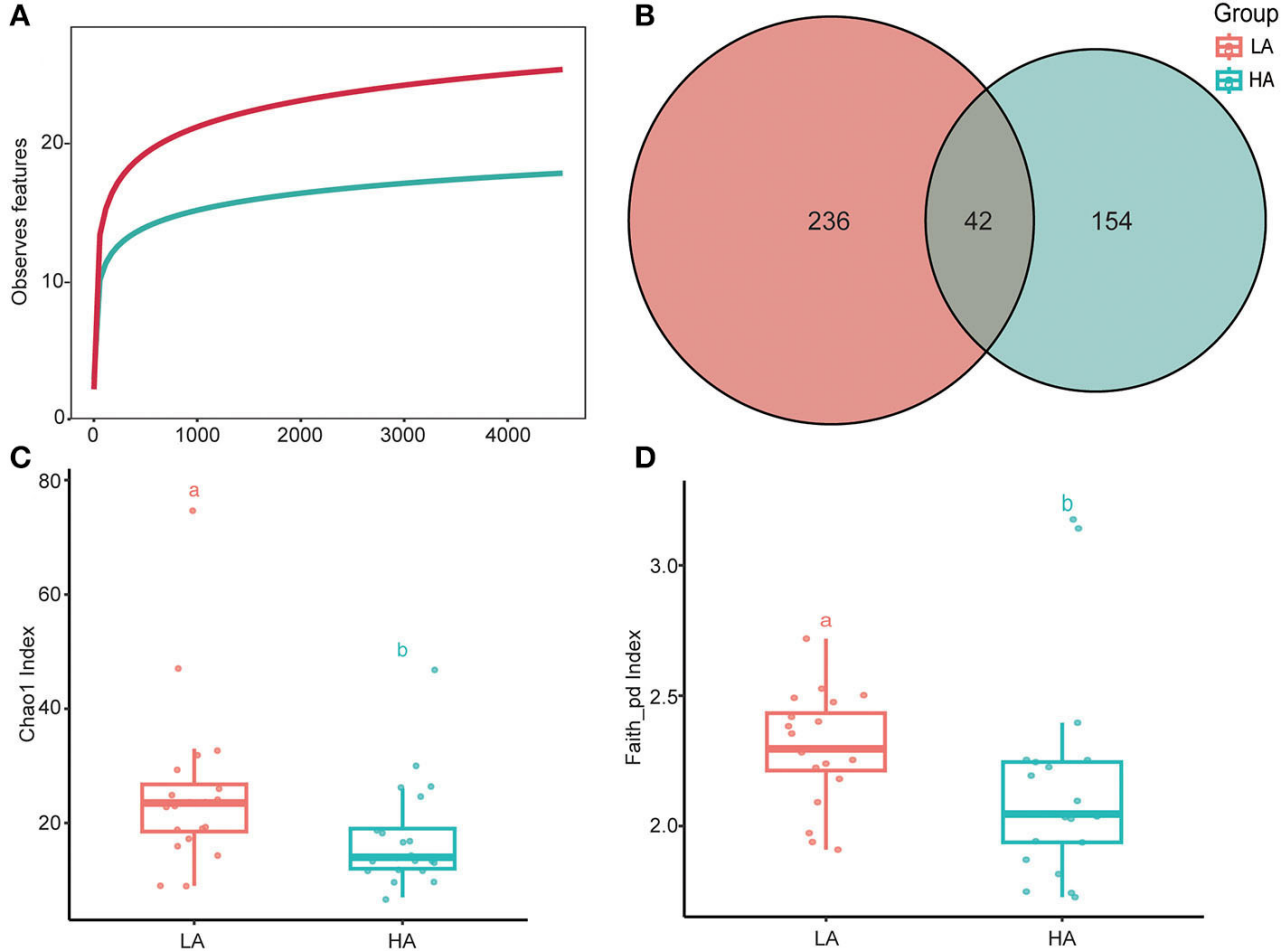
Alpha diversities of the gut microbiota differ between the HA (high-altitude, *n* = 21) and the LA (low-altitude, *n* = 20). **(A)** Rarefaction curves of detected bacterial ASVs of HA and LA; **(B)** A Venn diagram based on ASVS of HA and LA; **(C)** Chao1 index of the gut microbiota between HA and LA (*P* < 0.05); **(D)** Faith_pd index of the gut microbiota between HA and LA (*P* < 0.05). a and b are methods of representation in statistics, and they represent significant differences.

**Figure 3:**
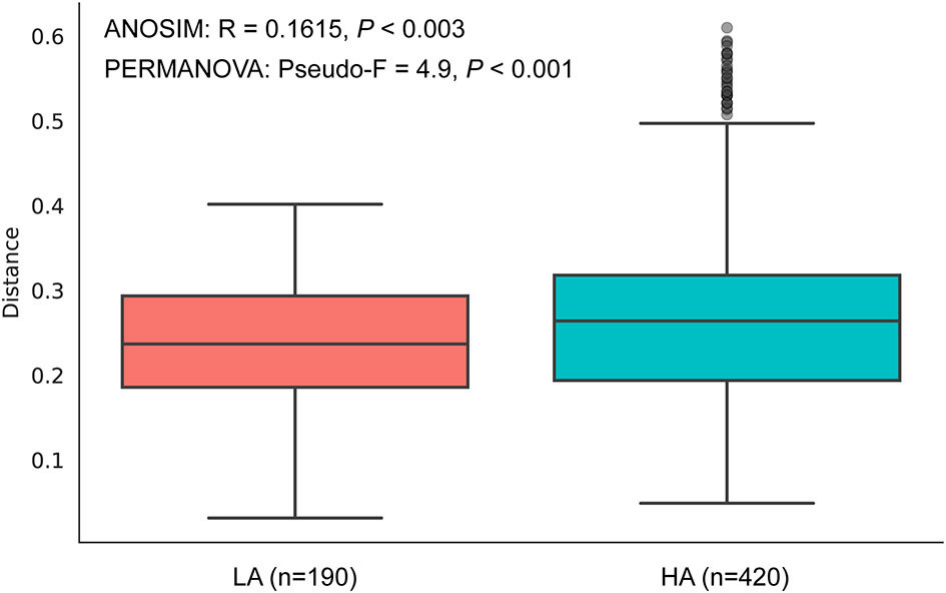
Beta diversities of the gut microbiota differ between the HA (high-altitude, *n* = 21) and the LA (low-altitude, *n* = 20) based on unweighted UniFrac distance. The horizontal axis represents the number of pairwise distances between intestinal individuals at HA and LA, respectively.

The rarefaction curves of bacterial ASVs were detected in the gut microbiota of high- and low-altitude bumblebees and reached a saturation phase by rarefying the samples to a minimum abundance of 4,530, which satisfied the subsequent analyses of gut microbiota alpha and beta diversity (Figure 2A). The two groups have 42 shared ASVs, with 154 unique ASVs at high altitude regions and 236 unique ASVs at low altitude regions (Figure 2B). The richness (Chao1) and phylogenetic diversity (faith_pd) of high-altitude bumblebee gut bacterial communities significantly decreased (*P* < 0.05) compared to those in low-altitude areas (Figures 2C, D). Analysis of similarities (ANOSIM) and permutational multivariate analysis of variance (PERMANOVA) were used to statistically test gut bacterial community similarities. The intestinal microbial community composition of bumblebees at high altitudes was significantly different from that in low-altitude areas (*P* < 0.05) (Figure 3).

### 3.2. Taxonomy difference of the gut microbiota of bumblebees between high- and low-altitude areas

**Figure 4:**
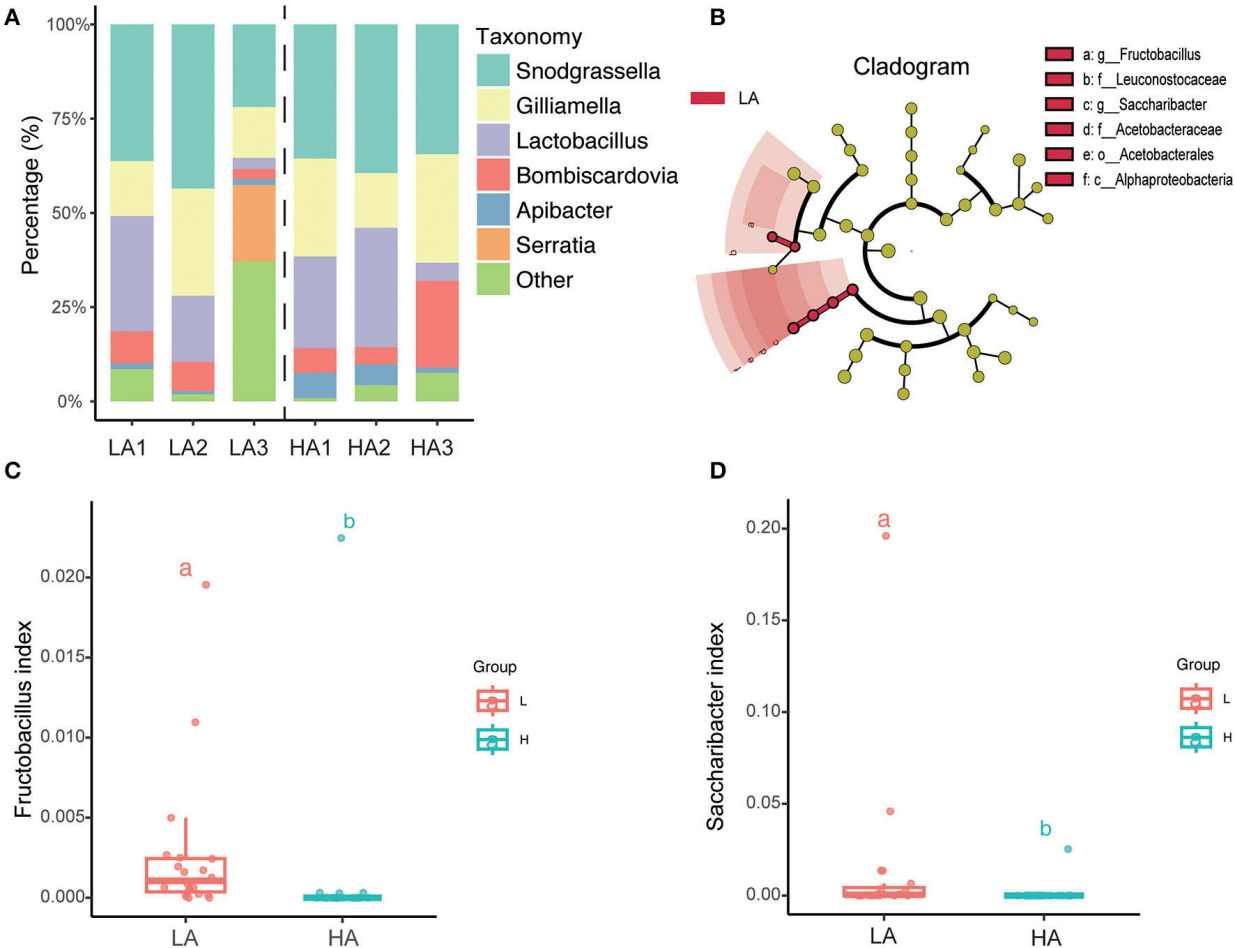
Variation of microbial taxonomy between the HA (high altitude, *n* = 21) and the LA (low altitude, *n* = 20). **(A)** Genus-level relative abundance of gut microbiota of the HA and LA; **(B)** LEfSe (LDA score > 2) analysis of the different classification hierarchy; **(C)** *Fructobacillus* relative abundance of gut microbiota at the HA and LA (*P* < 0.05); **(D)** *Saccharibacter* relative abundance of gut microbiota at the HA and LA (*P* < 0.05). a and b are methods of representation in statistics, and they represent significant differences.

The top six average relative abundances of the gut microbiota of *B. pyrosoma* are the core symbiotic bacteria *Snodgrassella, Lactobacillus, Gilliamella, Bombiscardovi, Apibacter*, and *Serratia* (Figure 4A). LEfSe (LDA score > 2) analysis of the different classification hierarchies showed that the gut community of high-altitude bumblebees has fewer Leuconostocaceae (Family), *Fructobacillus* (Genus), Alphaproteobacteria (Class), Acetobacterales (Order), Acetobacteraceae (Family), and *Saccharibacter* (Genus) compared with that of low-altitude bumblebees (Figure 4B). Furthermore, the boxplot showed that the bumblebee gut at high-altitude regions had less *Fructobacillus* (*P* < 0.05) and *Saccharibacter* (*P* < 0.05) compared to that at low-altitude regions (Figures 4C, D).

### 3.3. KEGG pathway difference in the gut microbiota of bumblebees between high- and low-altitude areas

**Figure 5:**
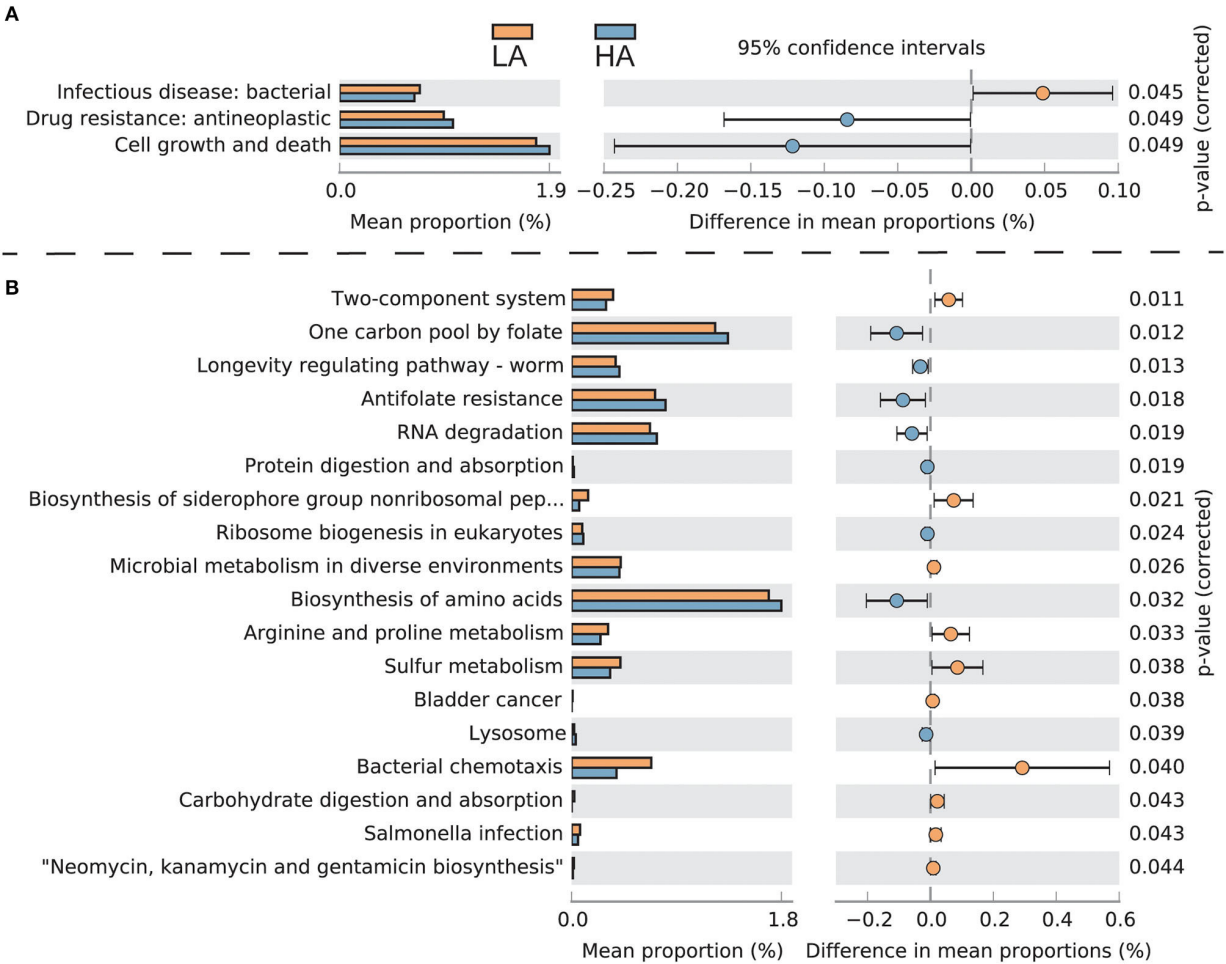
Functionally predicted KEGG pathways differing in gut microbiota between HA (high-altitude, *n* = 21) and LA (low-altitude, *n* = 20). The difference in proportions between the two groups is shown with 95% confidence intervals. Only a *P*-value of <0.05 (FDR adjusted) is shown and composition. **(A)** Secondary-level KEGG pathways; **(B)** Third-level KEGG pathways.

The intestinal microbes of bumblebees at high altitudes had three significant KEGG secondary classification pathways compared with those at low altitudes (*P* < 0.05). The gut microbiota of bumblebees at high altitudes has a greater number of pathways involving “Drug resistance: antineoplastic” and “Cell growth and death,” while the gut microbiota of bumblebees in low-altitude areas has a greater number of “Infectious disease: bacterial” (Figure 5A). For third-level KEGG pathways, the gut microbiota of bumblebee individuals in high-altitude regions exhibits a higher abundance of pathways related to “One carbon pool by folate,” “Antifolate resistance,” “Protein digestion and absorption,” and “Biosynthesis of amino acids.” Conversely, these high-altitude regions show a lower abundance of pathways associated with “Carbohydrate digestion and absorption” and “Salmonella infection” compared to the gut microbiota of bumblebees in low-altitude regions (Figure 5B).

## 4. Discussion

Compared with bumblebees in low-altitude regions, the gut bacteria of *B. pyrosoma* in high-altitude areas have lower alpha diversity and a relatively uniform community composition, which may result from collecting only a single type of plant pollen and nectar. Apart from host attributes (e.g., species, age, and caste) determining the gut microbiota of bumblebees, selective forces (e.g., antibiotic-resistant genes and habitat ecological environment) may also play an important role in shaping the formation of the gut microbial community (Colman et al., 2012; Kwong et al., 2014; Kwong and Moran, 2015, 2016; Zhang et al., 2021). For example, differences in external environments lead to different microbial community structures in wild and artificially reared bee colonies, with wild colonies having more non-core microbial groups (Zhang and Zheng, 2022). Owing to the obvious difference in eco-climate between high- and low-altitude areas, low temperature, hypoxia, ultraviolet radiation, and other climatic characteristics in low-altitude areas lead to the decline of plant diversity (Bruns and Kennedy, 2009; Murray, 2016). However, plant diversity is higher in low-altitude areas, where the gut microbiota displays higher diversity and has a more dispersed community composition.

The high-altitude regions have fewer *Fructobacillus* and *Saccharibacter*. It may be because there are fewer carbon sources at higher altitudes. The gut microbiota, such as *Fructobacillus* and *Saccharibacter*, generally benefit the health of their hosts. Members of the genus *Frugibacillus* are classified as frugiophilic lactic acid bacteria (Endo et al., 2018). The fructose content found in the nectar of quince cultivars from undamaged varieties was higher than that from cultivars found under frozen conditions (Akšić et al., 2014). To adapt to fructose-rich environments such as fruit and honey-rich flowers, *Fructobacillus* underwent a specific reductive evolution similar to that of *Lactobacillus kunkeei* (Lee et al., 2015; Maeno et al., 2016). *Frutobacillus* and *Saccharibacter*, present in the bumblebee gut, help the host degrade fructose through carbohydrate catabolism (Praet et al., 2018). The genus *Saccharibacter* is an acetic acid bacterium that has been shown to be associated with a variety of insects that rely on a high-sugar diet (Crotti et al., 2010; Chouaia et al., 2014). Since the genus *Saccharibacter* is isolated from flowers, it may also come into contact with insect pollinators and play a role in their use of nectar as a food source (Smith et al., 2020). However, some individuals in low-altitude areas even have a predominance of opportunistic bacteria such as *Serratia*, which may indicate that bumblebees in low-altitude areas face more severe environments such as ecological pollution.

Plateau-adapted hosts have developed strategies to cope with adverse environments, such as lowering their overall metabolic rate to reduce the body's demand for oxygen (Moore, 2017). Under low temperatures and hypoxic conditions, insects upregulate the expression of immune-related genes, such as those responsible for encoding antimicrobial peptides and the activation of the IKK/NF-kB signaling pathway, which in turn stimulates the immune response (Zhang et al., 2011; Xu and James, 2012; Bandarra et al., 2014). Compared to gut bacteria of bumblebee individuals in low-altitude areas, those in high-altitude regions have a higher abundance of pathways associated with “One carbon pool by folate,” “Antifolate resistance,” “Protein digestion and absorption,” and “Biosynthesis of amino acids.” Conversely, there is a decrease in pathways related to “Carbohydrate digestion and absorption” and “Salmonella infection.” This variation may be due to adverse environmental conditions such as low temperatures, hypoxia, and pollen and nectar food shortages at high altitudes. The Tibetan *B. pyrosoma* reduces energy metabolism and enhances immunity, which necessitates fewer carbohydrates and more protein to cope with these challenges. Correspondingly, the gut microbiota regulates members' species and abundance (e.g., *Fructobacillus* and *Saccharibacter*), exerting less demand for carbohydrates and improving protein utilization to help the host cope with an adverse high-altitude environment.

## 5. Conclusion

The gut microbiota co-evolved with their hosts for over 80 million years. Intestinal bacteria expand the host's ecological range through their metabolic capacities. Similarly, the host's ecological environment can also affect the composition and abundance of intestinal bacteria. Hosts at high altitudes, such as *Bombus pyrosoma*, adapt to the plateau environment by reducing energy metabolism and improving immunity. In turn, the gut microbiota helps the host adapt to the adverse environment by regulating their members' abundance and functional metabolism, such as reducing carbohydrate degradation and improving the utilization of protein. Our findings provide insights into the interactions between the bumblebee gut microbiota and the host.

## Data availability statement

The PacBio circular consensus sequencing (CCS) reads have been deposited in the Genome Sequence Archive in the BIG Data Center, Beijing Institute of Genomics (BIG), Chinese Academy of Sciences, under accession numbers CRA010937 that are publicly accessible at http://bigd.big.ac.cn/gsa, accessed on 7 May 2023.

## Author contributions

ZheZ and FY conceived the research project. ZheZ, MZ, FL, ZX, ZhiZ, HZ, YW, and JH collected the samples, analyzed the data, and sequenced the samples. ZheZ, YG, and JL wrote the manuscript. All authors read and approved the final version of the manuscript.

## References

[B1] Akšić M. F. Tosti T. Nedić N. Marković M. Ličina V. Milojković-Opsenica D.. (2014). Influence of frost damage on the sugars and sugar alcohol composition in quince (Cydonia oblonga Mill.) floral nectar. Acta. Physiologiae Plantarum 37. 10.1007/s11738-014-1701-y

[B2] An J. Huang J. Shao Y. Zhang S. Wang B. Liu X.. (2014). The bumblebees of North China (Apidae, Bombus Latreille). Zootaxa 3830, 1–89. 10.11646/zootaxa.3830.1.1 25081273

[B3] An J. Yao J. Huang J. Shao Y. Wu J. Li J.. (2008). *Bombus fauna* (Hymenoptera, Apidae) in Shanxi, China. Acta. Zootaxonomica Sinica, 33, 80–88.

[B4] Bandarra D. Biddlestone J. Mudie S. Muller H. A. Rocha S. (2014). Hypoxia activates IKK-NF-kappaB and the immune response in Drosophila melanogaster. Biosci. Rep. 34, e00127. 10.1042/BSR20140095 24993778 PMC4114064

[B5] Bruns T. D. Kennedy P. G. (2009). Individuals, populations, communities and function: the growing field of ectomycorrhizal ecology. New Phytol. 182, 12–14. 10.1111/j.1469-8137.2009.02788.x 19291070

[B6] Brutsaert T. D. (2007). Population genetic aspects and phenotypic plasticity of ventilatory responses in high altitude natives. Respir. Physiol. Neurobiol. 158, 151–160. 10.1016/j.resp.2007.03.004 17400521

[B7] Callahan B. J. Wong J. Heiner C. Oh S. Theriot C. M. Gulati A. S.. (2019). High-throughput amplicon sequencing of the full-length 16S rRNA gene with single-nucleotide resolution. Nucleic Acids Res. 47, e103. 10.1093/nar/gkz569 31269198 PMC6765137

[B8] Chang F. He S. Dang C. (2022). Assisted selection of biomarkers by linear discriminant analysis effect size (LEfSe) in microbiome data. J. Vis. Exp. e61715. 10.3791/61715 35635468

[B9] Chouaia B. Gaiarsa S. Crotti E. Comandatore F. Degli Esposti M. Ricci I.. (2014). Acetic acid bacteria genomes reveal functional traits for adaptation to life in insect guts. Genome Biol. Evol. 6, 912–920. 10.1093/gbe/evu062 24682158 PMC4007555

[B10] Colman D. R. Toolson E. C. Takacs-Vesbach C. D. (2012). Do diet and taxonomy influence insect gut bacterial communities? Mol. Ecol. 21, 5124–5137. 10.1111/j.1365-294X.2012.05752.x 22978555

[B11] Crotti E. Rizzi A. Chouaia B. Ricci I. Favia G. Alma A.. (2010). Acetic acid bacteria, newly emerging symbionts of insects. Appl. Environ. Microbiol. 76, 6963–6970. 10.1128/AEM.01336-10 20851977 PMC2976266

[B12] Douglas G. M. Maffei V. J. Zaneveld J. R. Yurgel S. N. Brown J. R. Taylor C. M.. (2020). PICRUSt2 for prediction of metagenome functions. Nat. Biotechnol. 38, 685–688. 10.1038/s41587-020-0548-6 32483366 PMC7365738

[B13] Endo A. Maeno S. Tanizawa Y. Kneifel W. Arita M. Dicks L.. (2018). Fructophilic lactic acid bacteria, a unique group of fructose-fermenting microbes. Appl. Environ. Microbiol. 84:e01290-18. 10.1128/AEM.01290-18 30054367 PMC6146980

[B14] Engel M. S. Rasmussen C. (2020). Corbiculate bees. Encyclopedia of Social Insects 10.1007/978-3-319-90306-4_30-1

[B15] Engel P. Moran N. A. (2013). Functional and evolutionary insights into the simple yet specific gut microbiota of the honey bee from metagenomic analysis. Gut Microbes 4, 60–65. 10.4161/gmic.22517 23060052 PMC3555888

[B16] Friedrich J. Wiener P. (2020). Selection signatures for high-altitude adaptation in ruminants. Anim. Genet. 51, 157–165. 10.1111/age.12900 31943284

[B17] Gao H. Chi X. Li G. Qin W. Song P. Jiang F.. (2020). Gut microbial diversity and stabilizing functions enhance the plateau adaptability of Tibetan wild ass (Equus kiang). Microbiologyopen 9, 1150–1161. 10.1002/mbo3.1025 32157819 PMC7294314

[B18] Hochachka P. W. Buck L. T. Doll C. J. Land S. C. (1996). Unifying theory of hypoxia tolerance: molecular/metabolic defense and rescue mechanisms for surviving oxygen lack. Proc. Natl. Acad. Sci. USA. 93, 9493–9498. 10.1073/pnas.93.18.9493 8790358 PMC38456

[B19] Huang J. X. An J. (2018). Species diversity, pollination application and strategy for conservation of the bumblebees of China. Biodivers. Sci. 26, 486–497. 10.17520/biods.2018068

[B20] Kanehisa M. Goto S. Sato Y. Furumichi M. Tanabe M. (2012). KEGG for integration and interpretation of large-scale molecular data sets. Nucleic Acids Res. 40, D109–D114. 10.1093/nar/gkr988 22080510 PMC3245020

[B21] Kwong W. K. Engel P. Koch H. Moran N. A. (2014). Genomics and host specialization of honey bee and bumble bee gut symbionts. Proc. Natl. Acad. Sci. USA. 111, 11509–11514. 10.1073/pnas.1405838111 25053814 PMC4128107

[B22] Kwong W. K. Medina L. A. Koch H. Sing K. W. Soh E. J. Y. Ascher J. S.. (2017). Dynamic microbiome evolution in social bees. Sci. Adv. 3,e1600513. 10.1126/sciadv.1600513 28435856 PMC5371421

[B23] Kwong W. K. Moran N. A. (2015). Evolution of host specialization in gut microbes: the bee gut as a model. Gut Microbes 6, 214–220. 10.1080/19490976.2015.1047129 26011669 PMC4615251

[B24] Kwong W. K. Moran N. A. (2016). Gut microbial communities of social bees. Nat. Rev. Microbiol. 14, 374–384. 10.1038/nrmicro.2016.43 27140688 PMC5648345

[B25] Lee F. J. Rusch D. B. Stewart F. J. Mattila H. R. Newton I. L. (2015). Saccharide breakdown and fermentation by the honey bee gut microbiome. Environ. Microbiol. 17, 796–815. 10.1111/1462-2920.12526 24905222

[B26] Liu Y. X. Qin Y. Chen T. Lu M. Qian X. Guo X.. (2021). A practical guide to amplicon and metagenomic analysis of microbiome data. Protein Cell 12, 315–330. 10.1007/s13238-020-00724-8 32394199 PMC8106563

[B27] Ma Y. Ma S. Chang L. Wang H. Ga Q. Ma L.. (2019). Gut microbiota adaptation to high altitude in indigenous animals. Biochem. Biophys. Res. Commun. 516, 120–126. 10.1016/j.bbrc.2019.05.085 31196622

[B28] Maeno S. Tanizawa Y. Kanesaki Y. Kubota E. Kumar H. Dicks L.. (2016). Genomic characterization of a fructophilic bee symbiont Lactobacillus kunkeei reveals its niche-specific adaptation. Syst. Appl. Microbiol. 39, 516–526. 10.1016/j.syapm.2016.09.006 27776911

[B29] Mockler B. K. Kwong W. K. Moran N. A. Koch H. (2018). Microbiome structure influences infection by the parasite crithidia bombi in bumble bees. Appl. Environ. Microbiol. 84, e02335-17. 10.1128/AEM.02335-17 29374030 PMC5861814

[B30] Moeller A. H. Foerster S. Wilson M. L. Pusey A. E. Hahn B. H. Ochman H. (2016). Social behavior shapes the chimpanzee pan-microbiome. Sci. Adv. 2, e1500997. 10.1126/sciadv.1500997 26824072 PMC4730854

[B31] Moore L. G. (2017). Measuring high-altitude adaptation. J. Appl. Physiol. (1985) 123, 1371–1385. 10.1152/japplphysiol.00321.2017 28860167 PMC5792094

[B32] Moran N. A. Ochman H. Hammer T. J. (2019). Evolutionary and ecological consequences of gut microbial communities. Annu. Rev. Ecol. Evol. Syst. 50, 451–475. 10.1146/annurev-ecolsys-110617-062453 32733173 PMC7392196

[B33] Murray A. J. (2016). Energy metabolism and the high-altitude environment. Exp. Physiol. 101, 23–27. 10.1113/EP085317 26315373

[B34] Nishida A. H. Ochman H. (2018). Rates of gut microbiome divergence in mammals. Mol. Ecol. 27, 1884–1897. 10.1111/mec.14473 29290090 PMC5935551

[B35] Parks D. H. Tyson G. W. Hugenholtz P. Beiko R. G. (2014). STAMP: statistical analysis of taxonomic and functional profiles. Bioinformatics 30, 3123–3124. 10.1093/bioinformatics/btu494 25061070 PMC4609014

[B36] Paulos S. Mateo M. De Lucio A. Hernandez-De Mingo M. Bailo B. Saugar J. M.. (2016). Evaluation of five commercial methods for the extraction and purification of DNA from human faecal samples for downstream molecular detection of the enteric protozoan parasites Cryptosporidium spp., Giardia duodenalis, and Entamoeba spp. J. Microbiol. Methods 127, 68–73. 10.1016/j.mimet.2016.05.020 27241828

[B37] Praet J. Parmentier A. Schmid-Hempel R. Meeus I. Smagghe G. Vandamme P. (2018). Large-scale cultivation of the bumblebee gut microbiota reveals an underestimated bacterial species diversity capable of pathogen inhibition. Environ. Microbiol. 20, 214–227. 10.1111/1462-2920.13973 29076622

[B38] Quast C. Pruesse E. Yilmaz P. Gerken J. Schweer T. Yarza P.. (2013). The SILVA ribosomal RNA gene database project: improved data processing and web-based tools. Nucleic Acids Res. 41, D590–D596. 10.1093/nar/gks1219 23193283 PMC3531112

[B39] Riek A. Stolzl A. Marquina Bernedo R. Ruf T. Arnold W. Hambly C.. (2019). Energy expenditure and body temperature variations in llamas living in the high andes of Peru. Sci. Rep. 9, 4037. 10.1038/s41598-019-40576-9 30858417 PMC6411917

[B40] Smith E. A. Vuong H. Q. Miller D. L. Parish A. J. Mcfrederick Q. S. Newton I. L. G. (2020). Draft genome sequences of four saccharibacter sp. strains isolated from native bees. Microbiol. Resour. Announc. 9, e00022-20. 10.1128/MRA.00022-20 32139579 PMC7171202

[B41] Storz J. F. Scott G. R. Cheviron Z. A. (2010). Phenotypic plasticity and genetic adaptation to high-altitude hypoxia in vertebrates. J. Exp. Biol. 213, 4125–4136. 10.1242/jeb.048181 21112992 PMC2992463

[B42] Weber R. E. (2007). High-altitude adaptations in vertebrate hemoglobins. Respir. Physiol. Neurobiol. 158, 132–142. 10.1016/j.resp.2007.05.001 17561448

[B43] Williams P. H. Huang J. Rasmont P. An J. (2016). Early-diverging bumblebees from across the roof of the world: the high-mountain subgenus Mendacibombus revised from species' gene coalescents and morphology (Hymenoptera, Apidae). Zootaxa 4204, 1–72. 10.11646/zootaxa.4204.1.1 27988613

[B44] Wu J. An J. D. Yao J. Huang J. X. Feng X. Q. (2009). *Bombus fauna* (Hymenoptera, Apidae) in Hebei, China. Acta. Zootaxonomica Sinica, 34, 87–97.

[B45] Xu J. James R. R. (2012). Temperature stress affects the expression of immune response genes in the alfalfa leafcutting bee, Megachile rotundata. Insect Mol. Biol. 21, 269–280. 10.1111/j.1365-2583.2012.01133.x 22356318

[B46] Yang J. Jin Z. B. Chen J. Huang X. F. Li X. M. Liang Y. B.. (2017). Genetic signatures of high-altitude adaptation in Tibetans. Proc. Natl. Acad. Sci. USA. 114, 4189–4194. 10.1073/pnas.1617042114 28373541 PMC5402460

[B47] Yu X. Wei P. Zhao S. Chen Z. Li X. Zhang W.. (2023). Population transcriptomics uncover the relative roles of positive selection and differential expression in Batrachium bungei adaptation to the Qinghai-Tibetan plateau. Plant Cell Rep. 42, 879–893. 10.1007/s00299-023-03005-w 36973418

[B48] Zhang J. Marshall K. E. Westwood J. T. Clark M. S. Sinclair B. J. (2011). Divergent transcriptomic responses to repeated and single cold exposures in Drosophila melanogaster. J. Exp. Biol. 214, 4021–4029. 10.1242/jeb.059535 22071194

[B49] Zhang Z. J. Huang M. F. Qiu L. F. Song R. H. Zhang Z. X. Ding Y. W.. (2021). Diversity and functional analysis of Chinese bumblebee gut microbiota reveal the metabolic niche and antibiotic resistance variation of Gilliamella. Insect Sci. 28, 302–314. 10.1111/1744-7917.12770 32101381

[B50] Zhang Z. J. Zheng H. (2022). Bumblebees with the socially transmitted microbiome: a novel model organism for gut microbiota research. Insect Sci. 29, 958–976. 10.1111/1744-7917.13040 35567381

[B51] Zheng H. Nishid,A A. Kwong W. K. Koch H. Engel P. Steele M. I.. (2016). Metabolism of toxic sugars by strains of the bee gut symbiont gilliamella apicola. mBio, 7, e01326-16. 10.1128/mBio.01326-16 27803186 PMC5090037

